# Cognitive Functioning in Rural Older Adults: The Mediating Role of Perceived Social Support

**DOI:** 10.3390/healthcare14020152

**Published:** 2026-01-07

**Authors:** Marko Krnjajić, Željko Mudri, Marija Barišić, Ivana Barać, Jasenka Vujanić, Maja Čebohin, Robert Lovrić, Katarina Major Poljak, Nikolina Farčić

**Affiliations:** 1 Department of Nursing and Palliative Medicine, Faculty of Dental Medicine and Health Osijek, Josip Juraj Strossmayer University of Osijek, Car Hadrijan Street 21, 31 000 Osijek, Croatia; m.krnjajic02@gmail.com (M.K.); mbarisic@fdmz.hr (M.B.); ibarac@fdmz.hr (I.B.); jvujanic@fdmz.hr (J.V.); mcebohin@fdmz.hr (M.Č.); 2 University Hospital Centre Osijek, Josip Huttler Street 4, 31 000 Osijek, Croatia; 3 Department of Sociology, Croatian Catholic University Zagreb, Ilica Street 242, 10 000 Zagreb, Croatia; 4 Medical Faculty of Osijek, Josip Huttler Street 4, 31 000 Osijek, Croatia; 5 Nursing Institute “Professor Radivoje Radić”, Faculty of Dental Medicine and Health Osijek, Josip Juraj Strossmayer University of Osijek, 31 000 Osijek, Croatia; rlovric@fdmz.hr; 6 Department of Integrative Dental Medicine, Faculty of Dental Medicine and Health Osijek, Josip Juraj Strossmayer University of Osijek, Crkvena Street 21, 31 000 Osijek, Croatia; kmajor@fdmz.hr

**Keywords:** aging, aged, older adults, cognitive function, cognitive disorders, social support, rural population, mediation analysis, cross-sectional studies, cognitive reserve

## Abstract

**Highlights:**

**What are the main findings?**

- Cognitive functioning and perceived social support in older adults differ significantly across age, educational attainment, marital status, and living arrangements.
- Perceived social support, particularly from friends, is strongly associated with better cognitive outcomes, while overall perceived social support mediates the observed association between living alone versus living with others and cognitive performance.

**What are the implications of the main findings?**

- Encouraging social support may help maintain cognitive functioning in older adults.
- Perceived social support mediates the relationship between living arrangement and cognitive functioning, highlighting its potential role in maintaining cognitive health in older adults.

**Abstract:**

**Background:** Aging is a multidimensional process influenced by biological, psychological, and social factors. Social support contributes to cognitive reserve by reducing stress, promoting mental engagement, and reinforcing a sense of belonging. **Objective:** To examine the association between perceived social support and cognitive functioning among older adults, and to investigate whether social support mediates the relationship between living arrangements and cognitive performance. **Methods:** The sample included 265 older adults aged 65 years and above, recruited using snowball sampling between March and July 2025 in rural communities. Instruments included the Montreal Cognitive Assessment for cognitive function and the Multidimensional Scale of Perceived Social Support for social support. **Results:** In this study, higher perceived social support from family, friends, and significant others was associated with better cognitive performance. Multiple regression showed that all three dimensions of social support significantly predicted MoCA scores, with the strongest effect from friends’ support. Mediation analysis revealed that perceived social support mediated the relationship between living arrangements and cognitive functioning, while the direct effect of cohabitation alone was not significant. **Conclusions:** These results highlight the importance of perceived social support in preserving cognitive health in older adults.

## 1. Introduction

Aging is a dynamic, complex and unavoidable process that involves biological, psychological and social changes. The course and intensity of these changes are largely influenced by individual characteristics, lifestyle habits as well as environmental and genetic factors [1]. Although everyone aspires to age well by maintaining functional capacity and quality of life in advanced age, this goal largely depends on preserving optimal cognitive and brain functioning [2]. Many older adults experience cognitive decline to varying degrees, which in some may progress to severe impairment and pathological conditions, while others retain most of their cognitive abilities [3]. Continuous cognitive engagement and challenging the adaptability and flexibility of the brain can help older adults mitigate or even reverse some of the negative consequences of age-related brain degeneration through neuroplasticity [4,5].

### 1.1. Cognitive Aging and Concept of Cognitive Reserve

The concept of cognitive reserve, developed by Yaakov Stern and colleagues, further explains individual differences in cognitive aging by postulating the existence of compensatory mechanisms within the brain [6]. A higher level of cognitive reserve is associated with improved cognitive functioning in older age, even in the presence of brain changes observed in Alzheimer’s disease and other dementias [7]. Individuals with a higher level of cognitive reserve are able to maintain cognitive functioning despite having smaller brain volumes, indicating that the advantages of cognitive reserve operate independently of structural brain differences [8]. Strengthening cognitive reserve therefore appears to play a role in delaying the onset of clinically significant cognitive decline, particularly because individuals with greater reserve enter later life with a higher baseline of cognitive functioning, enabling them to remain above impairment thresholds for a longer period even when the rate of decline does not differ between individuals [9]. Lifelong intellectual, educational, occupational, and social activities contribute to resilience against degenerative brain changes, while factors such as educational attainment, professional involvement, participation in complex mental activities, social support and community connectedness play an important role in maintaining this reserve [10]. Within the context of social support, engagement in socially enriching environments offers cognitive stimulation that may help preserve cognitive functioning even in the presence of structural brain deterioration [11].

### 1.2. Role of Social Support in Maintaining Cognitive Reserve

Social support contributes to the maintenance of cognitive reserve, particularly given the changing nature of social roles and interactions during aging. It encompasses the help, protection, and sense of belonging that individuals receive through reciprocal social networks and relationships characterized by trust and emotional closeness [12,13]. Moreover, social connections may buffer the effects of brain atrophy on cognition, as individuals with smaller brain volumes can maintain cognitive performance when engaged in diverse and stimulating social networks [14]. Older adults with larger and more diverse social networks tend to perform better on cognitive assessments [15].

Engagement in varied social contexts requires individuals to manage multiple roles and interactions, a cognitive challenge that may strengthen cognitive reserve. Additionally, older adults often experience changes in their social roles and interactions due to physical, emotional, social, or environmental factors. These changes may involve losses, adaptations, and new experiences that shape how older individuals perceive and maintain their social ties [13].

Conversely, individuals embedded in dense, homogenous networks experience less cognitive stimulation, as most contacts occupy similar roles and are interconnected [14]. Reciprocal social relationships and the amount of both received and provided support have a positive impact on overall health and quality of life in older adults, including disease prevention, fewer physical health problems, longer life expectancy, improved cognitive abilities, greater self-esteem, and a sense of belonging [16]. Importantly, the benefits of social support for cognitive reserve are not determined solely by the size or diversity of one’s network. Rather, the quality of relationships, characterized by emotional closeness, trust, and meaningful engagement, is crucial for maintaining cognitive function and overall well-being in older adults [17].

Given the limited effectiveness of pharmacological interventions in preventing or slowing cognitive aging [18], research has increasingly focused on non-pharmacological factors that may support cognitive health. Among these, social support has been shown to play an important role, as it can enhance cognitive reserve and help maintain cognitive function in older adults [19].

### 1.3. Demographic Decline, Population Ageing and Structural Vulnerability in Rural Vukovar-Srijem County

Vukovar-Srijem County is characterized by markedly adverse demographic trends, including below-average population density, a negative migration balance, and accelerated population ageing [20]. The severity of these changes is reflected in the steady annual decrease of approximately 2.5 percent in the rural population, accompanied by a substantial increase in the number of residents aged 65 years and older [21]. This demographic divergence of simultaneous depopulation and ageing intensifies structural vulnerability in rural communities. Rural areas are also additionally burdened by geographical remoteness, insufficient transport infrastructure, and limited availability of social and healthcare services, all of which reduce the mobility of older adults and hinder their social integration. Under such conditions, maintaining social networks becomes particularly challenging, as older adults face growing isolation while the younger population continues to migrate [22]. According to estimates, only about 5 percent of older adults living in rural areas of the Republic of Croatia use formal long-term healthcare services, which is substantially below the European average of 8 percent [23]. Consequently, older adults rely to a considerable extent on unpaid and private care, which may adversely affect their cognitive and psychosocial well-being in the rural communities of Vukovar-Srijem County [24]. In the context of depopulation, population ageing, and infrastructural limitations, understanding the role of social support is essential for planning interventions aimed at improving the well-being of older adults. However, evidence remains scarce regarding how perceived social support operates in relation to cognitive functioning among older adults living in structurally vulnerable rural environments, where demographic decline, limited service availability, and fragmented family networks may fundamentally alter both the availability and the role of social support.

The objective of this study was to examine the association between perceived social support and cognitive functioning in older adults, and to determine whether social support mediates the relationship between living arrangements and cognitive performance.

## 2. Materials and Methods

### 2.1. Study Design

This quantitative, cross-sectional study was conducted between March and July 2025 in rural communities of Vukovar-Srijem County, Croatia. To reduce anxiety and increase participant comfort, data were collected in participants’ private homes. This approach has been shown to enhance the reliability of cognitive testing in familiar environments [25].

### 2.2. Participants

The study included older adults aged 65 years and above residing in rural areas who were able to provide informed consent. Exclusion criteria were age below 65 years, residence in urban communities, and living in institutionalized care facilities.

A non-probabilistic snowball sampling method was used to recruit 265 participants who met the inclusion criteria. The estimated sampling error was 6% with a 95% confidence interval, calculated using an online sample size calculator [26]. Given the lack of prior confidential relationships with many participants, chain-referral (snowball) sampling was employed, whereby each participant was asked to identify other potential respondents meeting the inclusion criteria, facilitating recruitment of individuals who might otherwise have been difficult to reach [27].

### 2.3. Instruments

The study employed three instruments: the Montreal Cognitive Assessment (MoCA), the Multidimensional Scale of Perceived Social Support (MSPSS), and a questionnaire collecting general and sociodemographic information (age, sex, marital status, place of residence, educational attainment, number of children and grandchildren). Participants completed the questionnaire using the paper-and-pencil method.

#### 2.3.1. Montreal Cognitive Assessment

MoCA is a screening tool for mild cognitive impairment, covering multiple cognitive domains, including executive functions, language, attention and concentration, conceptual thinking, calculation, orientation, memory, and visuoconstructive abilities. The total possible score is 30, with scores of 26 or above considered normal [28]. Scoring below 26 does not necessarily indicate the presence of cognitive impairment. Training and certification for the administration, scoring, and interpretation of the MoCA were completed online in accordance with standard requirements to ensure accuracy and for the purposes of this study. The Croatian version was translated by Dr. I. Martinić Popović and used with the permission of the original author, Dr. Ziad Nasreddine. The internal consistency of MoCA in this study was 0.766.

The psychometric properties of the MoCA have been extensively examined across multiple studies employing diverse methodological approaches. Lam et al. [29] reported that the MoCA demonstrates good diagnostic performance in clinical samples, with good sensitivity, specificity, positive and negative predictive values, and overall accuracy. Compared with other screening instruments, such as the Rowland Universal Dementia Assessment Scale, the MoCA has shown superior sensitivity and accuracy for the detection of mild cognitive impairment and dementia, with performance remaining stable across different levels of education, first language, and race [30]. Swaminathan et al. [31] reported that, based on receiver operating characteristic (ROC) analyses, the MoCA outperformed the DCTclock™ in discriminating between cognitively impaired and unimpaired individuals and in identifying Alzheimer’s disease-related neuropathology. Pinto et al. [32] found that the MoCA was more sensitive than the Mini-Mental State Examination for tracking mild cognitive impairment and Alzheimer’s disease. Sala et al. [33] have additionally confirmed a stable hierarchical factorial structure, strong general factor saturation, and measurement invariance across age, education, socioeconomic status, and gender, supporting the MoCA’s validity as a measure of global cognition in older adults. Overall, these findings from previous research support the MoCA as a reliable and valid cognitive screening tool for use among heterogeneous older adult populations.

#### 2.3.2. Multidimensional Scale of Perceived Social Support

MSPSS consists of 12 items assessing perceptions of social support adequacy across three domains: family, friends, and significant others, with four items per domain. A meta-analytic confirmatory factor analysis of 57 unique studies, conducted by Koğar and Koğar confirmed the three-factor structure, supporting its theoretical division [34]. It is among the most widely translated and psychometrically validated instruments for measuring perceived social support [35]. It specifically addresses the subjective assessment of social support adequacy [36], therefore reflecting perceived rather than objective or structural support. Despite its subjective nature and the potential for socially desirable responding, previous studies have demonstrated the validity of the MSPSS. Specifically, nonsignificant correlations between both total MSPSS and its subscale scores and the Marlowe-Crowne Social Desirability Scale indicate that high MSPSS scores cannot be attributed solely to social desirability bias [37]. Findings of confirmation studies suggest that factors other than social desirability account for the reported levels of perceived social support, even in diverse samples [37], making the MSPSS an appropriate instrument for the present study. Responses are rated on a 7-point Likert scale from 1 (“strongly disagree”) to 7 (“strongly agree”). Subscale scores (family, friends, significant others) and the total score are calculated by averaging the relevant items. The summed total score ranges from 12 to 84, while the mean total score ranges from 1 to 7. Higher scores indicate greater perceived social support [38]. This instrument was used with permission from the author, Gregory D. Zimet. While Dambi et al., in a systematic review of the psychometric properties, acknowledged variability in the robustness of translated MSPSS versions [35], the version used in the present study demonstrated excellent internal consistency: Cronbach’s α = 0.905 for the total scale, α = 0.970 for the Family subscale, α = 0.942 for the Friends subscale, and α = 0.992 for the Significant Other subscale.

### 2.4. Data Collection Procedure

Prior to data collection, participants received a research notice form describing the study purpose, confidentiality, voluntary participation, and the right to withdraw at any time. Researchers also explained these details verbally and confirmed participants’ understanding before they signed the informed consent form. Participants first completed the self-administered MSPSS questionnaire. Afterward, the researcher conducted the MoCA cognitive assessment. For participants with 10–12 years of formal education, one point was added to the total MoCA score to adjust for educational attainment, in accordance with established recommendations [39,40,41]. Finally, general and demographic information was collected, as personal questions could influence responses [42]. In self-administered sections, researchers provided clarifications in a neutral tone of voice without suggesting or directing responses. Completion time was not limited. The average time to complete the full assessment, including cognitive testing, self-assessment of perceived social support, and collection of general and demographic information, was 30 min per participant, ranging from 20 to 50 min.

### 2.5. Ethical Considerations

The study was conducted in accordance with the ethical principles of the Declaration of Helsinki. Participation was voluntary and anonymous, and participants could withdraw at any time. Written approval was obtained from the Higher Institution Ethical Committee (Class: 602-01/25-12/03; IRB: 2158/97-97-10-25-12). All participants were informed, both in writing (through a research notice form) and verbally, about the study objectives, ethical aspects, and instructions for completing the questionnaire. Participation was voluntary, confirmed by signing the informed consent form. Anonymity was guaranteed by separating the questionnaire from the consent form. Respondents were informed that they could withdraw from the study at any time.

### 2.6. Data Analysis

The frequency distributions of the variables were described using descriptive statistical methods. The distribution of numerical variables was assessed with the Shapiro–Wilk test, which indicated a statistically significant deviation from normality for the variables under study (*p* < 0.05). Measures of central tendency and dispersion were expressed as medians (Me) and interquartile ranges (IQR) due to the non-normal distribution of the data. The Mann–Whitney U test was used to examine differences between two independent groups, and the Kruskal–Wallis test with Bonferroni correction was applied for comparisons among three or more groups. Effect size was reported using the rank-biserial correlation (r_rb_) [43]. Multiple linear regression analysis was conducted to assess whether perceived social support predicts cognitive functioning. Mediation analysis was performed to examine whether perceived social support mediates the relationship between living arrangements and cognitive functioning. Due to the underrepresentation of certain living arrangements (e.g., spouse and children: n = 20, 7.5%; spouse, children, and grandchildren: n = 19, 7.2%; with child/children: n = 20, 7.5%; with child/children and grandchildren: n = 8, 3%), the variable of living arrangement was dichotomized for the mediation analysis. Specifically, it was coded as “living alone” versus “living with others in the household” to ensure adequate subgroup sizes and statistical power [44]. Prior to the analyses, regression assumptions were checked, confirming the absence of multicollinearity and influential outliers, and verifying the distribution of residuals. Statistical significance was set at *p* < 0.05. All analyses were performed using JASP software, version 0.19.3 (Department of Psychological Methods, University of Amsterdam, Amsterdam, the Netherlands).

## 3. Results

A total of 265 participants from rural areas of Vukovar-Srijem County took part in the study, the majority of whom were female (n = 166; 62.6%). Regarding the level of educational attainment, most participants had completed primary school (n = 179; 67.5%). More than half of the respondents were married (51.3%), while 44.2% were widowed. Most participants lived either alone (38.9%) or with their spouse in a nuclear family (35.8%). The majority had children (89.1%) and grandchildren (82.3%). The median age of the participants was 72 years (IQR = 68–75) (Table 1).

### 3.1. Differences in Total, Family, Friends and Significant Other Perceived Social Support

#### 3.1.1. Educational Attainment

Participants with a secondary educational attainment reported significantly higher total perceived social support, family support, and friend support (*p* < 0.001, r_rb_ = 0.375; *p* < 0.001, r_rb_ = 0.375; *p* < 0.001, r_rb_ = 0.335; medium effect sizes) compared to those with only primary education. Participants with undergraduate/graduate educational attainment also reported higher total, family, and friend support (*p* = 0.037, r_rb_ = 0.423; *p* = 0.037, r_rb_ = 0.423; *p* = 0.013, r_rb_ = 0.482) than participants with primary education. Higher support from other significant persons was perceived in participants with secondary education compared to those with primary education (*p* < 0.001, r_rb_ = 0.324; medium effect size) (Table 2).

#### 3.1.2. Marital Status and Living Arrangement

Married participants reported higher total perceived social support than widowed (*p* < 0.001, r_rb_ = 0.757), single (*p* < 0.001, r_rb_ = 0.958), and divorced participants (*p* = 0.005, r_rb_ = 0.941), all representing large effect sizes. Married participants also reported higher family support than widowed (*p* < 0.001, r_rb_ = 0.318; medium effect size) and single participants (*p* < 0.001, r_rb_ = 0.842; large effect size), and higher support from significant others than widowed (*p* < 0.001, r_rb_ = 0.839), single (*p* < 0.001, r_rb_ = 0.997), and divorced participants (*p* = 0.023, r_rb_ = 0.949; large effect sizes). Widowed participants reported higher family support than single participants (*p* = 0.029, r_rb_ = 0.696; large effect size), and married participants reported higher friend support than widowed participants (*p* = 0.005, r_rb_ = 0.243; small effect size).

Participants living in nuclear families (with a spouse) reported higher total perceived social support than those living alone (*p* < 0.001, r_rb_ = 0.783; large effect size) and those in multigenerational households (*p* = 0.021, r_rb_ = 0.230; small effect size). Participants in multigenerational households reported higher total support than those living alone (*p* < 0.001, r_rb_ = 0.505; medium effect size). Higher family, friend, and significant others’ support was reported by participants in nuclear families (*p* < 0.001, r_rb_ = 0.371; medium; *p* = 0.008, r_rb_ = 0.243; small; *p* < 0.001, r_rb_ = 0.867; large) and multigenerational households (*p* < 0.001, r_rb_ = 0.340; medium; *p* = 0.003, r_rb_ = 0.299; small; *p* < 0.001, r_rb_ = 0.462; medium) compared to those living alone. Nuclear family participants reported higher support from significant others than those in multigenerational households (*p* < 0.001, r_rb_ = 0.343; medium effect size) (Table 2).

#### 3.1.3. Parental Status and Grandparent Status

Participants with children reported higher total perceived social support (*p* < 0.001, rrb = 0.426; medium effect size) and family support (*p* < 0.001, rrb = 0.584; large effect size) than participants without children. Participants with grandchildren reported higher total perceived social support (*p* = 0.006, r_rb_ = 0.254; small effect size) and family support (*p* < 0.001, r_rb_ = 0.397; medium effect size) than those without grandchildren (Table 2).

#### 3.1.4. Chronological Age and Gender

Participants aged 65–74 years (*p* = 0.008, r_rb_ = 0.614; large effect size) and 75–84 years (*p* = 0.004, r_rb_ = 0.589; large effect size) reported significantly higher perceived friend support than participants aged 85 years and older. Additionally, male participants reported higher perceived social support from significant others (*p* = 0.003, r_rb_ = 0.215; small effect size) (Table 2).

Measures of central tendency indicate an overall lower level of cognitive functioning in the sample, with a median total MoCA score of 20 (IQR = 17–24). Participants with a secondary educational attainment (*p* < 0.001, r_rb_ = 0.701; large effect size) and those with undergraduate/graduate education (*p* < 0.001, r_rb_ = 0.790; large effect size) demonstrated significantly higher cognitive functioning compared to participants with only primary education. Married participants exhibited significantly higher cognitive functioning than widowed participants (*p* < 0.001, r_rb_ = 0.384; medium effect size). Participants living in a nuclear family (with a spouse) (*p* < 0.001, r_rb_ = 0.341; medium effect size) and those living in multigenerational households (*p* < 0.001, r_rb_ = 0.373; medium effect size) also showed significantly higher cognitive functioning. Participants with children had significantly higher cognitive functioning compared to those without children (*p* = 0.002, r_rb_ = 0.349; medium effect size). Finally, participants aged 65–74 years demonstrated significantly higher cognitive functioning than those aged 75–84 years (*p* < 0.001, r_rb_ = 0.694; large effect size) and those aged 85 years and older (*p* < 0.001, r_rb_ = 0.410; medium effect size) (Table 3).

**Figure 1:**
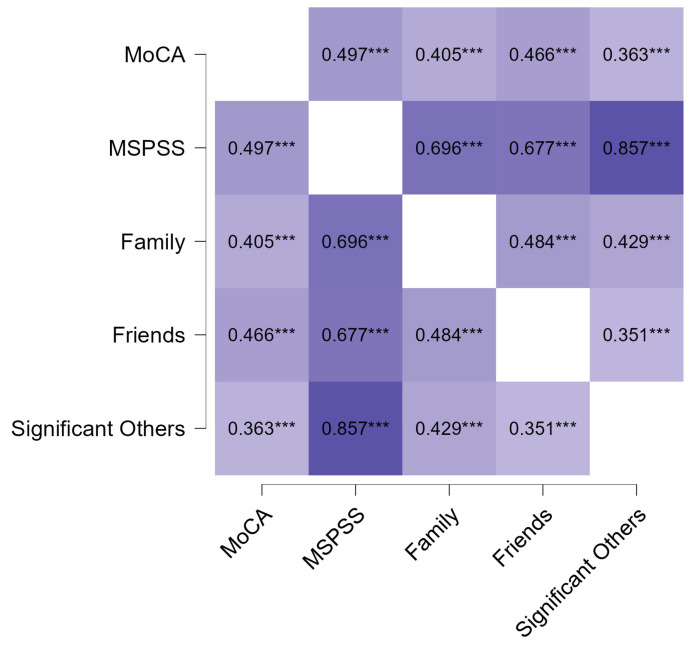
Spearman correlation heatmap between perceived social support and total cognitive functioning. *** *p* < 0.001. All correlations are statistically significant and positive, with darker colors representing higher correlation values and lighter colors representing lower correlation values.

A statistically significant positive association was observed between the total MoCA score and levels of perceived social support, including its subscales. Higher levels of total perceived social support (ρ = 0.497; *p* < 0.001), family support (ρ = 0.405; *p* < 0.001), friend support (ρ = 0.466; *p* < 0.001), and support from other significant persons (ρ = 0.363; *p* < 0.001) were associated with higher MoCA scores (Figure 1).

Participants with normal cognitive functioning reported significantly higher levels of perceived social support compared to those with impaired cognitive functioning, including total perceived social support (r_rb_ = 0.569), family support (r_rb_ = 0.536), friend support (r_rb_ = 0.582), and support from other significant persons (r_rb_ = 0.423), all representing large effect sizes (Table 4).

The model included three independent variables (subscales of social support: family, friends, and significant others). The model was statistically significant (F(3, 261) = 35.583, *p* < 0.001) and explained 29% of the total variance in MoCA scores (R^2^ = 0.290, Adjusted R^2^ = 0.282). All three dimensions of social support were significant predictors of MoCA scores, with the strongest contribution from friends’ support (β = 0.371, *p* < 0.001), while family support (β = 0.160, *p* = 0.007) and support from significant others (β = 0.162, *p* = 0.004) also had a significant positive effect. Higher perceived social support was associated with better cognitive functioning (Table 5).

Significant positive correlations were found between perceived social support and cognitive functioning (r = 0.497, *p* < 0.001), and between living arrangement, social support (r = 0.452, *p* < 0.001), and cognitive functioning (r = 0.292, *p* < 0.001) (Table A1).

**Figure 2:**
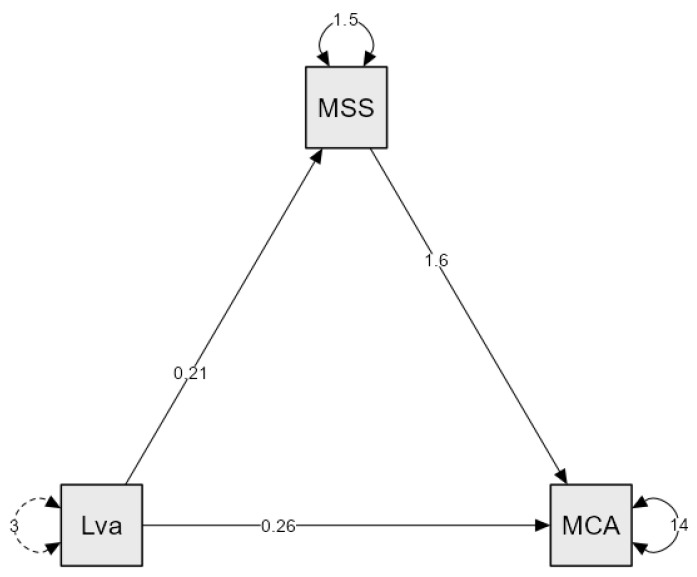
Mediation model of Perceived Social Support on Cognitive Functioning. MSS—MSSP; Lva—Living arrangement; MCA—MoCa. Dotted arrows indicate indirect (mediation) effects; solid arrows indicate direct effects.

The direct effect of living arrangement on cognitive functioning was positive but not statistically significant (β = 0.258, *p* = 0.062), suggesting a trend toward a positive association. The indirect effect, mediated by perceived social support, was β = 0.332 (*p* < 0.001; 95% CI [0.177, 0.487]), confirming a significant mediation. Individuals living with someone reported higher levels of perceived social support, which in turn contributed to better cognitive functioning. The total effect of living arrangement on the MoCA score was β = 0.589 (*p* < 0.001), indicating that living arrangement has a significant positive impact on cognitive functioning, primarily through the effect of social support (Table 6, Figure 2).

## 4. Discussion

The results of the conducted study indicate that older adults represent a heterogeneous population in which social and cognitive outcomes differ across factors such as age, gender, educational attainment, marital status, and family structure. These findings underscore the importance of considering both social and structural determinants when examining cognitive outcomes in later life, as emphasized by Livingston et al. [45]. In this context, social support was examined in structurally vulnerable environments characterized by demographic decline, limited service availability, and fragmented family networks, which may fundamentally alter both its availability and role. Social support may vary between urban and rural populations due to differences in community structure, social networks, and access to resources. Jones et al. [46] found that rural participants reported higher levels of perceived social support compared to their urban counterparts. This distinction is relevant for interpreting the present findings, as the availability and perceived adequacy of social support could be influenced by the rural context in which participants live, potentially moderating its relationship with cognitive outcomes.

Regarding participants’ characteristics, most participants were aged 65–74, whereas adults aged 85 and older were underrepresented. Such a distribution is common in healthcare and gerontological research, as the oldest-old are less likely to participate due to health, mobility, or functional limitations [47]. Additionally, it was observed that older adults with higher educational attainment perceive greater social support, which is consistent with previous studies [48]. It could be postulated that older adults with lower educational attainment may have smaller or less active social networks, as lower educational attainment has frequently been associated with social isolation [49]. Furthermore, higher educational attainment influences older adults’ opportunities to access social activities, participate, and achieve better social integration, and it also enables them to make more effective use of community resources [50]. Therefore, individuals with higher educational attainment are expected to exhibit better cognitive function, largely due to the enhancement of cognitive reserve through lifelong intellectual, educational, occupational, and social activities [10]. This is also because individuals with greater cognitive reserve enter later life with a higher baseline level of cognitive functioning [9].

The observed low median MoCA score, indicating an overall lower level of cognitive functioning in the sample, is consistent with the known sensitivity of MoCA to educational attainment. This aligns with Kistler-Fischbacher et al. [51], who reported that median MoCA scores vary according to age and education, with older participants and those with fewer years of education showing lower median scores. The relatively low proportion of participants with higher education in our sample may have influenced the observed prevalence of cognitive impairment and effect sizes related to educational attainment. Despite applying an education-adjusted scoring procedure [adding one point for participants with ≤12 years of education] [39], participants with lower educational levels were more likely to score below the standard MoCA cut-off. Thus, the observed MoCA scores likely reflect an interplay between cognitive functioning and educational background rather than cognitive impairment alone.

In addition to marital status, family roles such as being a parent or grandparent also contribute to perceived social support. Married older adults perceive higher levels of social support and achieve better cognitive outcomes, consistent with Sommerlad et al. [52], who report that being married is associated with healthier lifestyle behaviors and lower mortality, and may reduce the risk of dementia due to life-course factors, as well as increased daily social interaction and support, which enhance cognitive reserve. In contrast, lower levels of perceived social support and cognitive functioning are more frequently observed among single and widowed older adults, likely due to the impact of social isolation and its association with adverse cognitive outcomes [53,54].

Older adults who are parents and/or grandparents perceive higher overall social support, particularly from family, which is consistent with previous findings [55]. Hou et al. [55] demonstrated that intergenerational support from adult children is positively associated with cognitive functioning in middle-aged and older adults, and may mediate the relationship between grandparenting and cognitive function. Thus, caring for grandchildren can indirectly contribute to cognitive functioning by fostering intergenerational support from adult children [55]. Frequent, high-quality interactions with children and grandchildren may contribute to emotional stability and help maintain cognitive abilities through social engagement and stimulation, supporting previous evidence on the role of social interactions in preserving cognitive function [55,56].

Older adults living in nuclear families reported higher levels of perceived social support, in line with previous research suggesting that social support may differ across living arrangements. In accordance with this, previous studies also indicate that the relationship between perceived social support and cognitive function may vary depending on living arrangements [57,58]. Social support from intergenerational relationships can provide older adults with a sense of belonging, purpose, and emotional fulfillment, which may reduce stress and anxiety [59,60]. In addition, these relationships offer opportunities for cognitive stimulation and learning, which can help maintain cognitive function and prevent age-related decline [57]. For example, Yu et al. [58] reported that, compared to older adults living with a spouse, those living alone, with adult children, with both spouse and adult children, or with others experienced faster cognitive decline. Moreover, the association between living arrangements and cognitive decline was gender-specific: living alone was linked to faster decline only in older men, whereas living with spouse and adult children or living with others was linked to faster decline only in older women [58]. Taken together, these findings suggest that perceived social support varies across living arrangements and may represent an important contextual factor through which differences in cognitive functioning observed in previous research can be interpreted.

A difference was also observed in the perception of social support from significant others among men. As the category of “significant other(s)” is left to the respondent to define [61], it is assumed that the significant other category most often includes their spouse, who is expected to care for their partner’s needs. Men tend to rely on their spouse for intimacy, emotional, instrumental, and caregiving support [50]. Al-Kandari [62] also states that having a living wife is an important factor for men’s health and well-being in general, as the wife is one of the major sources of social support for older adult men.

Although gender differences in overall MoCA scores were not significant in the present study, previous research indicates that women often perform better in verbal domains, as highlighted in methodological evaluations of the MoCA [28]. Beyond gender, age-related differences were also observed, with the oldest-old reporting lower levels of perceived social support. Lower perceived levels of social support among the oldest-old may reflect a decrease in social interactions and the size of social networks as age increases [63]. Since social participation has been found to decrease with age in both women and men, it is hypothesized that perceived social support also declines alongside reduced social engagement, particularly in women, although this gender difference diminishes after the age of 80 [50]. In contrast, Lara et al. [53] and Fjell et al. [64] report that women, on average, have broader and more functional social networks and utilize social support more effectively as a protective mechanism, although this was not observed in the present study. It could be hypothesized that this is due to sociocultural specificities, as Plužarić et al. [65] in the context of connectedness with family and friends, did not find any significant gender differences among older adults in Croatia.

Age has consistently been identified as a key predictor of cognitive decline, as reported by Piolatta et al. [16], and the results of this study align with these findings, with older participants achieving lower MoCA scores. The positive association between perceived social support and cognitive functioning is consistent with previous evidence [19,66] linking social support and cognitive activity to a reduced risk of subsequent cognitive impairment. Subjective feelings of social support and social integration may benefit cognitive functioning, particularly in stressful situations, by reducing stress and lowering levels of stress hormones such as cortisol, which has been shown to negatively affect cognitive performance [67].

Additionally, the highest correlation and the greatest explained variance in cognitive functioning were observed within the friend support subscale, which may be explained by the fact that friendships often encourage participation in social and cognitively stimulating activities and enhance older adults’ sense of belonging [56]. Because maintaining friendships has been shown to require more active effort and engagement in shared activities, activity engagement may be an underlying pathway explaining the distinct associations between contact frequency with friends versus family and cognition [56]. Friendship ties also play a uniquely protective role in later-life cognitive functioning. Maintaining or restoring active friendship networks promotes social engagement and cognitive stimulation, with the recovery of previously lost friendships being particularly beneficial for cognitive functioning, especially among older men [68]. Therefore, individuals who perceive higher levels of support from family, friends, or significant others may experience better cognitive outcomes, as social engagement provides emotional, instrumental, and cognitive stimulation that helps preserve cognitive functioning. The results also highlight the importance of the perceived level of social support, rather than merely the absence of social isolation.

Mediation analysis suggests that living with someone is associated with higher cognitive functioning primarily through perceived social support, rather than through the mere number of cohabitants. This conceptualization of perceived social support as a potential mediator is based on theoretical models that view it as a psychosocial pathway connecting structural social conditions with cognitive and health-related outcomes. Berkman et al. [69] describe the impact of social networks as a cascading process, spanning macro-social to psychobiological factors, which dynamically interact to influence health outcomes. They also define social support as a primary pathway through which social networks may influence physical and mental health status, while noting that it is not the only relevant factor. These theoretical considerations align with empirical results reported by Amieva et al. [70], highlighting the importance of the quality and perception of social relationships in maintaining cognitive abilities in older adults. Participants who felt satisfied with their relationships had a 23% lower risk of dementia, and those who reported giving less support than they received over their lifetime had a 55% lower risk of dementia and a 53% lower risk of Alzheimer’s disease, respectively. Importantly, the only variables associated with subsequent dementia or Alzheimer’s disease were those reflecting the quality of relationships [70].

Similarly, Glei et al. showed that, despite a social structure in which older adults frequently live with their children and social interactions are predominantly family-centered, participation in social activities outside the family had a stronger association with cognitive functioning than contacts with family members or non-relatives [71]. These findings further support the proposed mediation role of perceived social support. Nevertheless, the observed mediation effect is statistical and does not imply temporal or causal relationships; it provides a theoretical basis for future longitudinal studies to explore whether perceived social support causally influences cognitive functioning. Given the inconsistent conclusions regarding the specific nature of the association between social support and cognitive functioning among older adults [72], alternative plausible causal directions should be acknowledged. Prior studies suggest that reverse causality and bidirectional relationships are possible, whereby better cognitive functioning may facilitate greater formal and informal social engagement [73]. Moreover, large panel studies using cross-lagged models indicate that declines in cognitive function may precede increased social isolation [74], while other longitudinal analyses suggest reciprocal influences in which social relationships and cognitive functioning mutually affect one another, with higher-quality social ties acting as a protective factor in cognitive aging [75]. Although causal inferences cannot be drawn due to the cross-sectional design, the findings point to perceived social support as a potentially modifiable component that may be targeted by interventions, pending confirmation in longitudinal and experimental studies.

### Strengths, Limitations and Implications for Future Research

This study has several notable strengths. First, it adopts a clear conceptual focus on perceived social support, distinguishing it from structural or objective social network characteristics. This approach aligns with theoretical models emphasizing subjective appraisal as a psychosocial mechanism influencing cognitive reserve. Second, the study addresses an underrepresented population by focusing on older adults living in structurally vulnerable rural communities characterized by demographic decline and limited access to formal services, thereby enhancing its public health relevance. Third, the use of validated instruments with excellent internal consistency supports the reliability of the findings. Finally, the inclusion of mediation analysis enables a more nuanced examination of perceived social support as a potential pathway linking living arrangements and cognitive functioning.

Despite the valuable findings, this study has several limitations. The cross-sectional design does not allow causal inferences, and longitudinal research is needed to clarify the direction of the association between perceived social support and cognitive abilities. This is particularly relevant in the context of the mediation model, whose effect is statistical and does not imply temporal or causal relationships, although it provides a theoretical basis for future studies to examine whether perceived social support causally influences cognitive outcomes. In addition, the current underrepresentation of certain living arrangements, which were dichotomized in the mediation model due to small sample sizes, should be addressed in future studies to enable more comprehensive analyses. In addition, the use of snowball sampling, along with other potentially influential factors such as participants’ physical and mental health status and the recruitment of predominantly socially active individuals, which were not controlled for, may have introduced selection bias and limited the representativeness of the sample. These aspects highlight key considerations that warrant further investigation in future research.

Furthermore, the instruments used carry methodological constraints: the MoCA is sensitive to educational attainment [76], while the MSPSS reflects a subjective perception of support that may not correspond to its actual structure or quality [38]. The sample was relatively homogeneous in geographical and cultural terms, which limits the generalizability of the findings, particularly given the known cultural variations in social support structures [77]. In addition, the participants were predominantly younger older adults compared to the oldest-old group, which may result in a somewhat more optimistic picture relative to the broader older population.

Nevertheless, the study highlights the importance of perceived social support for cognitive functioning in older adults and points to key directions for future research. Future studies would benefit from longitudinal designs and the use of randomized or probability-based sampling strategies to enhance representativeness and generalizability. In addition, a combination of quantitative and qualitative approaches, the inclusion of diverse cultural contexts, and the development of intervention models aimed at enhancing social engagement are warranted. Particular attention should be given to assessing the influence of digital forms of social support, which are increasingly relevant for improving social support among older adults [78].

## 5. Conclusions

The present study demonstrates that older adults in rural areas of Vukovar-Srijem County represent a heterogeneous population, with cognitive functioning and perceived social support varying across key sociodemographic and family-related characteristics. Higher levels of perceived social support and better cognitive functioning were observed among participants with higher educational attainment, those who were married, those with children, and those living in nuclear or multigenerational households. Perceived social support was positively associated with cognitive functioning across all dimensions, with support from friends emerging as the strongest predictor of cognitive performance. Mediation analysis indicated that the association between living arrangements and cognitive functioning is largely accounted for by perceived social support, suggesting that the quality of social relationships may be more relevant for cognitive health than cohabitation itself.

From a theoretical perspective, the findings support cognitive reserve theory and models positioning perceived social support as a pathway linking social environments to cognitive aging. From a practical perspective, the findings underscore the importance of interventions aimed at enhancing social support networks, promoting social engagement, and fostering meaningful interpersonal relationships in rural communities. Such measures may help mitigate the negative effects of ageing and limited access to formal care, ultimately supporting cognitive health and overall well-being in older adults.

## Figures and Tables

**Table 1:** Sociodemographic characteristics of respondents.

Characteristics of Respondents	Category	n (%)
Gender	Male	99 (37.4)
	Female	166 (62.6)
	Other	0 (0)
Educational attainment	Primary school	179 (67.5)
	Secondary school	74 (27.9)
	Undergraduate/graduate degree	12 (4.5)
Marital status	Married	136 (51.3)
	Widowed	117 (44.2)
	Divorced	5 (1.9)
	Never married	7 (2.6)
Living arrangement	Living alone	103 (38.9)
	Nuclear family (spouse)	95 (35.8)
	Spouse and children	20 (7.5)
	Spouse, children and grandchildren	19 (7.2)
	With child/children	20 (7.5)
	With child/children and grandchildren	8 (3)
Parental status	Yes	236 (89.1)
	No	29 (10.9)
Grandparent status	Yes	218 (82.3)
	No	47 (17.7)
Chronological age	65–74 years	186 (70.2)
	75–84 years	69 (26)
	≥85 years	10 (3.8)
	Me (IQR) = 72 (68–75)	

**Table 2:** Differences in Perceived Social Support (Total, Family, Friends, Significant Other) Among Older Adults by General Characteristics—MSPSS.

Characteristics of Respondents		MSPSS (Total)	*p* Value	Family	*p* Value	Friends	*p* Value	Significant Other	*p* Value
		Me (IQR)		Me (IQR)		Me (IQR)		Me (IQR)	
Gender	Male	5.58 (4.33–6.25)	0.070	6.00 (5.13–7.00)	0.463	5.00 (4.00–5.63)	0.619 *	6.00 (3.00–7.00)	0.003 *
	Female	4.66 (4.00–6.00)		6.50 (5.81–7.00)		5.00 (4.25–5.75)		4.88 (1.00–6.19)	
Educational attainment	Primary school	4.67 (3.92–5.83)	<0.001 †	6.00 (5.00–7.00)	0.003 †	5.00 (3.75–5.25)	<0.001 †	5.00 (1.00–6.00)	<0.001 †
	Secondary school	5.88 (4.48–6.50)		6.88 (6.00–7.00)		5.00 (4.56–6.00)		6.00 (2.44–7.00)	
	Undergraduate/graduate degree	6.08 (4.58–6.56)		6.88 (6.69–7.00)		5.75 (5.00–6.25)		6.13 (3.00–6.63)	
Marital status	Married	6.00 (5.33–6.50)	<0.001 †	7.00 (6.00–7.00)	<0.001 †	5.00 (4.50–6.00)	0.004 †	6.00 (6.00–7.00)	<0.001 †
	Widowed	4.25 (3.58–4.67)		6.00 (5.00–7.00)		5.00 (3.75–5.00)		1.00 (1.00–3.00)	
	Divorced	4.08 (4.00–4.17)		5.50 (5.00–6.50)		4.50 (2.75–5.00)		3.00 (2.00–3.00)	
	Never married	2.67 (2.33–4.00)		3.00 (2.50–5.00)		5.00 (4.00–5.50)		1.00 (1.00–1.00)	
Living arrangement	Living alone	4.08 (3.38–4.67)	<0.001 †	6.00 (5.00–6.75)	<0.001 †	4.75 (3.00–5.00)	<0.001 †	1.00 (1.00–3.00)	<0.001 †
	Nuclear family (spouse)	5.92 (5.33–6.38)		7.00 (6.00–7.00)		5.00 (4.50–6.00)		6.00 (6.00–7.00)	
	Multigenerational family	5.25 (4.33–6.42)		7.00 (6.00–7.00)		5.00 (4.50–6.00)		5.50 (1.00–7.00)	
Parental status	Yes	5.17 (4.31–6.25)	<0.001 *	6.75 (6.00–7.00)	<0.001 *	5.00 (4.25–5.75)	0.085 *	5.25 (1.00–6.50)	0.222 *
	No	3.83 (2.92–5.33)		5.00 (2.50–6.00)		5.00 (3.00–5.00)		2.50 (1.00–6.50)	
Grandparent status	Yes	5.17 (4.27–6.23)	0.006 *	6.75 (6.00–7.00)	<0.001 *	5.00 (4.25–5.75)	0.272 *	5.13 (1.00–6.44)	0.817 *
	No	4.33 (3.46–5.79)		6.00 (4.75–6.13)		5.00 (3.38–5.00)		3.00 (1.00–7.00)	
Chronologicalage	65–74 years	5.17 (4.25–6.31)	0.126	6.50 (5.81–7.00)	0.127 †	5.00 (4.25–6.00)	0.005 †	5.63 (1.00–7.00)	0.061 †
	75–84 years	4.58 (3.83–5.83)		6.00 (5.00–7.00)		5.00 (4.25–5.50)		4.25 (1.00–6.00)	
	85 years and older	5.00 (4.75–5.56)		6.00 (5.13–6.69)		3.63 (3.00–4.13)		5.63 (5.13–6.00)	

* Mann–Whitney U test; † Kruskal–Wallis test; Note: Higher perceived social support was consistently observed among married participants, those living with a spouse, and respondents with higher educational attainment, particularly within the family and significant other subscales. Participants with children and those with grandchildren reported higher levels of total and family social support. Higher support from friends was observed among younger participants (those in early and middle old age), while male participants reported higher perceived support from significant others.

**Table 3:** Differences in Total MoCa Score According to General Characteristic.

Variable	Category	MoCA	*p*-Value
		Median (Interquartile Range)	
Gender	Male	21 (18–21)	0.140 *
	Female	20 (17–20)	
Level of education	Primary school	19 (16–21)	<0.001 †
	Secondary school	25 (22–27)	
	Undergraduate/graduate degree	25.5 (22.75–28)	
Marital status	Married	22 (19–25)	<0.001 †
	Widowed	18 (16–22)	
	Divorced	21 (20–21)	
	Never married	19 (14–21.50)	
Living arrangement	Living alone	18 (15–22)	<0.001 †
	Nuclear family (spouse)	21 (18.50–25)	
	Multigenerational households	22 (19–25)	
Parental status	Yes	21 (18–24)	0.002 *
	No	17 (15–21)	
Grandparent status	Yes	20.5 (17.25–24)	0.064 *
	No	20 (15.5–23)	
Chronological age	65–74 years	21 (18–25)	<0.001 †
	75–84 years	18 (16–21)	
	≥85 years	15 (14.25–16.75)	

* Mann–Whitney U test; † Kruskal–Wallis test; Note: There were significant differences in cognitive functioning across levels of education, marital status, living arrangements, parental status, and age. Participants with higher education, married, living in nuclear or multigenerational households, with children, and younger in age had higher median MoCA scores, with effect sizes ranging from medium to large.

**Table 4:** Differences in Perceived Social Support According to the Cut-off for a Normal MoCA Score.

Variable	Cognitive Functioning Category	Me (IQR)	*p*-Value *
Total score of MSPSS	Normal	6.33 (6.00–6.67)	<0.001
	Impaired	4.71 (4.00–5.83)	
Family	Normal	7.00 (7.00–7.00)	<0.001
	Impaired	6.00 (5.25–7.00)	
Friends	Normal	6.00 (5.00–6.00)	<0.001
	Impaired	5.00 (3.81–5.25)	
Significant Other	Normal	7.00 (6.00–7.00)	<0.001
	Impaired	5.00 (1.00–6.00)	

* Mann–Whitney U test.

**Table 5:** Multiple Linear Regression of MoCA Total Score on MSPSS Subscale.

Predictor	Unstandardized B	Standardized β Coefficient	t	*p*	95% CL
Intercept	10.557	-	9.449	<0.001	[8.357, 12.756]
Family	0.521	0.160	2.719	0.007	[0.144, 0.898]
Friends	1.193	0.371	6.332	<0.001	[0.822, 1.564]
Significant Other	0.285	0.162	2.907	0.004	[0.092, 0.478]

β = standardized regression coefficient; R^2^ for the model = 0.290.

**Table 6:** Mediation Analysis of Living Arrangement on Cognitive Functioning Through Perceived Social Support.

Effect	Path	ß	SE	95% CL	z	*p*-Value
Total	Living arrangement * → MoCa	0.589	0.149	[0.297, 0.881]	3.954	<0.001
Indirect	Living arrangement → MSSP →MoCa	0.332	0.079	[0.177, 0.487]	4.196	<0.001
Direct	Living arrangement → MoCa	0.258	0.138	[−0.013, 0.528]	1.867	0.062

* Living arrangement as a dummy variable (Living alone vs. living with others in household); Delta method standard errors, ML estimator.

## Data Availability

The original data presented in the study are openly available on FigShare at https://doi.org/10.6084/m9.figshare.30705632 (accessed on 31 December 2025).

## Appendix A

**Table A1:** Spearman Correlations Between Perceived Social Support, Total Cognitive Functioning and Living arrangement.

Variable	1	2
1. Total score of MSSP	-	-
2. Total score of MoCa	0.497 *	-
3. Living arrangement	0.452 *	0.292 *

* *p* < 0.001.
